# Complex Tasks Force Hand Laterality and Technological Behaviour in Naturalistically Housed Chimpanzees: Inferences in Hominin Evolution

**DOI:** 10.1100/2012/514809

**Published:** 2012-04-01

**Authors:** M. Mosquera, N. Geribàs, A. Bargalló, M. Llorente, D. Riba

**Affiliations:** ^1^Universitat Rovira i Virgili (URV), Campus Catalunya, Avinguda de Catalunya 35, 43002 Tarragona, Spain; ^2^Institut Català de Paleoecologia Humana i Evolució Social (IPHES), Campus Catalunya, Avinguda de Catalunya 35, 43002 Tarragona, Spain; ^3^Unitat de Recerca i Laboratori d'Etologia, Fundació Mona, Carretera de Cassà 1 km, Riudellots de la Selva, 17457 Girona, Spain

## Abstract

Clear hand laterality patterns in humans are widely accepted. However, humans only elicit a significant hand laterality pattern when performing complementary role differentiation (CRD) tasks. Meanwhile, hand laterality in chimpanzees is weaker and controversial. Here we have reevaluated our results on hand laterality in chimpanzees housed in naturalistic environments at Fundació Mona (Spain) and Chimfunshi Wild Orphanage (Zambia). Our results show that the difference between hand laterality in humans and chimpanzees is not as great as once thought. Furthermore, we found a link between hand laterality and task complexity and also an even more interesting connection: CRD tasks elicited not only the hand laterality but also the use of tools. This paper aims to turn attention to the importance of this threefold connection in human evolution: the link between CRD tasks, hand laterality, and tool use, which has important evolutionary implications that may explain the development of complex behaviour in early hominins.

## 1. Introduction

Hand laterality is a cognitive factor according to which a group of individuals (populations or species) differentially use one hand (left or right) to perform a task [1] or a group of tasks [2]. From a behavioural point of view, the importance of hand laterality lies in the fact that in humans it is the most developed functional asymmetry. Hand laterality seems to be an indicator of brain hemispheric specialisation, which is not exclusive to humans. It is present in species such as rats (*Rattus norvegicus*) [3], elephants (*Elephas maximus*) [4], humpback whales (*Megaptera novaeangliae*) [5], and crows (*Corvus macrorhynchos*) [6]. Actually, Rogers [7] suggests that all vertebrates share brain hemispheric specialisation. However, brain hemispheric specialisation seems to be also related in humans to linguistic functions. Therefore, its pattern of emergence and development throughout human evolution can provide insight into the evolution of human cognitive capacities.

In modern humans, 97% of the population is hand lateralised, and between 85% and 90% of individuals are right-handed [8]. However, several studies have found great diversity in the expression of hand laterality [9–17], which appears to be influenced by environmental and cultural factors [18] and by the motor actions involved in performing the task at hand [19]. Despite this variability, research in non-Western societies confirms the universality of hand laterality in the species *Homo sapiens* [20]. Results from three preindustrial cultural groups—the G/wi (Botswana), Himba (Namibia), and Yanomamo (Venezuela)—show right-hand dominance at the population level for all tasks and stronger preferences for conducts involving tools. Even when discounting the strong biases of Western educative influences [21], the pattern of right-handedness in modern humans emerges. This has led to the widely accepted belief that human hand laterality may be conditioned by biological factors [8, 22] with inheritable components [23, 24].

Therefore, most research suggests the existence of a *genetic component* for hand preference, although neither the inherited pattern nor the responsible gene or genes have yet been identified [25–27]. Two main genetic models [22, 28] propose that hand laterality and brain dominance for language depend on a single gene with two alternative alleles. Both models assume that the gene for laterality is unique and exclusive to human beings. However, some studies on chimpanzees contradict this suggestion.

Research on hand laterality in nonhuman primates has been conducted for decades. The aim of these studies is to understand how and when hand laterality was fixed into the evolutionary history of our order. Copious data have been gathered regarding the hand laterality of *Pan*, *Gorilla*, and *Pongo*; however, no clear manual tendencies have been identified. The most abundant data come from studies on chimpanzees, because such animals are more easily accessible and frequently make and use tools both in the wild and in captivity [29, 30].

There are two opposing positions concerning hand laterality in chimpanzees. One position supports right-hand dominance in chimpanzees, given its high incidence (67%) among this species [31]. The other position rejects this manual asymmetry at the population level [2, 32, 33]. These differences are mainly due to different conceptions concerning empirical studies and conflicting viewpoints at the theoretical level [2, 31].

Despite these divergences, some overall tendencies can be observed regarding hand laterality in nonhuman primates. Firstly, nonhuman primates display clear evidence of lateralisation at the individual level. Secondly, they show population asymmetries for some behaviours, particularly complex and structured behaviours. Thirdly, differences between human and nonhuman primates seem to be of more degree than nature, that is, weaker laterality is seen in the latter. Beside interspecies differences, the main disparity of results seems to be related to the living environment of the samples studied (wild or in captivity) and the type of tasks performed (simple or complex).

Therefore, hand laterality in humans has proved to be universal, while hand laterality at the population level in nonhuman primates remains controversial. However, hypotheses on the emergence of hand laterality are based on nonhuman primate studies. Several factors have been suggested as the cause of this emergence, such as body posture, bipedalism, tool use, and task complexity. The primary difference between the hypotheses proposed is the emphasis given to one factor as the key element around which the others turn.

To begin with, the *postural origin hypothesis* [34] stresses the importance of body posture in facilitating right-hand dominance for handling objects from a primate arboreal ancestor. On the other hand, the *bipedalism hypothesis *[35–37] suggests that the emergence of hand dominance in humans developed from bipedal posture, through the improvement of the brain skills needed to keep the body balanced in this stance. This hypothesis is supported by several studies with nonhuman primates [36, 38–42]. Additionally, the advent of bipedalism may have favoured the development of different tasks performed by the upper limbs, such as gesture communication or the use of tools [43].

Thirdly, the *tool use hypothesis* argues that hand dominance evolved because of the bimanual coordination required in making and using tools. Therefore, the strong manual asymmetry of the genus *Homo* would be the product of the systematic manufacture and use of tools [44–46]. This hypothesis is also supported by several studies with nonhuman primates [29, 47–51].

Finally, the *task complexity hypothesis *[52] considers that hand laterality depends on the nature of the tasks to be performed. Low-level tasks demand low cognitive and motor involvement, so they are poor indicators of hand and brain lateralisation. In contrast, high-level tasks call for precise motor actions and cognitive complexity, so they are good indicators of manual and brain lateralisation. This hypothesis has been empirically supported by several studies with nonhuman primates [51, 53–55]. Actually, it seems that the *task complexity hypothesis *complements both the *tool use hypothesis *and the *bipedalism hypothesis*, since complexity increases both when a vertical position is adopted and when instruments are used.

Uomini [56] has recently published a study that supports the *task complexity hypothesis* for the emergence of hand preference. She proposes that only tasks involving complementary role differentiation (CRD) [57] are indicative of hand laterality. A task of this type requires the action of both hands performing different roles. In contrast, coordinated bimanual tasks are those in which both hands play the same role. CRD tasks are also known as bimanual complementary (see [2] for definition) and bimanual complex tasks [58].

In her study, Uomini [56] conducted two experiments to test handedness in humans. In the first experiment, several people were asked to refit fragments of a flint core. In this task both hands were active, but performing the same role. In the second experiment, the same people were asked to crack nuts, which involved both hands in different roles. As a consequence of this difference, when performing the flint refitting, individuals did not show significant hand laterality, whereas, during the nut-cracking task, hand laterality was evident. The author aimed to demonstrate that, when humans are asked to do the same experiments as chimpanzees, only bimanual CRD tasks, as opposed to coordinated bimanual tasks, are significant indicators of handedness, despite extreme human hand laterality. In our view, this conclusion is extremely important and has implications regarding both hand lateralisation and human evolution that must be further studied.

In light of Uomini's results [56], we have revisited the results of our studies on hand laterality in chimpanzees housed in naturalistic environments. Uomini's research shows that, although hand laterality in humans has been widely proved, it can be as complex and variable as in nonhuman primates. Only CRD tasks appear to express clear hand laterality in humans. In accordance with this assertion, we have reevaluated our results on hand laterality in naturalistically housed chimpanzees [49, 51], with special attention to CRD tasks. In this paper, we present a review of these results from an evolutionary perspective. The chimpanzees from our sample appeared to show a link between CRD tasks, hand laterality, and technological behaviour that may provide insight into the development of complex technological behaviour in early hominins.

## 2. Hand Laterality and Tool Use in Naturalistically Housed Chimpanzees

Research on chimpanzee hand laterality yields contradictory results depending on whether it is conducted in the wild [59, 60] or in captivity [61–63]. It has been argued that these differences are not solely due to the environment but to the different tasks studied as well [64]. Therefore, we performed our research on chimpanzees sheltered in two naturalistic environments—Fundació Mona in Spain and Chimfunshi Wildlife Orphanage in Zambia—and we studied different types of tasks, from unimanual spontaneous tasks to CRD bimanual tasks.

### 2.1. Fundación Mona

Fundació Mona (FM) (Riudellots de la Selva, Girona, north-eastern Spain) (41° 54′ N, 2° 49′ E) (www.fundacionmona.org) was opened in the year 2000 and is devoted to the rescue, rehabilitation, and sheltering of primates that have been exploited or mistreated. Today, FM shelters a group of chimpanzees (*Pan troglodytes*) made up of 10 males and 3 females, ranging from 6 to 53 years old. (See Table  1 in [49], for additional information about age, classes, sex, and rearing history of each individual.)

The institution consists of a naturalistic outdoor enclosure of 5,640 m^2^ and two socialisation enclosures of 25 m^2^ connected to a pavilion measuring 140 m^2^. The outdoor enclosure has natural ground with Mediterranean and riverside vegetation. Several structures made of wood, rope, and nets, as well as a shallow pond, have been built in this enclosure. Water supply is readily available, and curators provide food four times a day. Juices, fresh fruits, special dehydrated food, fresh vegetables, boiled rice, nuts, and seeds complete the chimpanzees' diet. This food is delivered in special containers or left on the ground. The enclosure is surrounded by a steel fence and a 12 V electrified fence.

Since 2000, three experiments have been performed to evaluate the handedness of FM chimpanzees: spontaneous tasks [65], simple reaching [49], and the hose task [49, 51].

Our first study at FM was an observational study [65]. Ten chimpanzees (8 males and 2 females) were observed while performing daily spontaneous tasks. The aim of this study was to detect hand preference in the chimpanzees at FM. 111 hours of data were recorded over a period of 11 months. The ethological methodology was based on other authors' works. The observational protocol followed the observational rules described by Altmann [66] and Martin and Bateson [67]. The behavioural catalogue was built on the catalogues described for wild chimpanzees [59, 60, 68]. Finally, the recording of the unimanual and bimanual tasks followed the procedures described in McGrew and Marchant [60].

**Figure 1:**
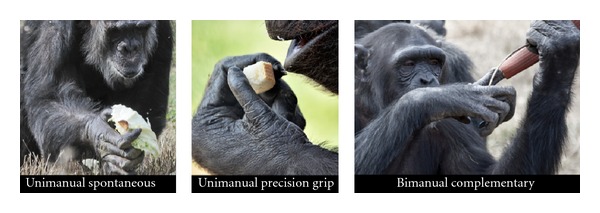
Different tasks performed by some of the chimpanzees at Fundació Mona (Girona, Spain): unimanual spontaneous task, unimanual with precision grip (simple reaching), and bimanual complementary task (hose task) with tool use. (Credit: Miquel Llorente.)

A total of 3,496 bouts were recorded. Results showed that 89.0% of the bouts (*n* = 3, 110) corresponded to unimanual tasks (Figure 1) and only 11.0% of the bouts (*n* = 386) corresponded to bimanual tasks. The latter were divided into “coordinated tasks” (96.6%) and to a much lesser extent “complementary tasks” or CRD tasks (3.4%). Three of the ten individuals displayed a statistically significant preference for the left hand, two individuals were on the significance borderline (one for left-hand preference and another for right-hand preference), while the other five individuals did not move significantly away from a chance selection of left or right hand. In terms of manual preferences according to activity, five individuals showed a statistically significant manual preference in some pattern, whereas the remainder showed no significant preference in any task. The one-sample *t*-test concluded that none of the activities studied in this work showed significant differences.

In summary, spontaneous tasks were mainly unimanual, and they did not lead to hand dominance either as a result of the activity or the individual. However, our current analyses with a wider sample are pointing to the existence of low degree of hand laterality at individual level for spontaneous unimanual tasks. In our view, this different result is related to the bigger size of the sample. We understand that much more data than the previously obtained was needed to detect this pattern. Similar results on unimanual and bimanual tasks have been achieved by other authors. Of the actions Marchant and McGrew [59] recorded at Gombe, 86% were unimanual and 14% bimanual. At Mahale, McGrew and Marchant [60] detected 87.4% unimanual actions and 12.6% bimanual actions, of which around 65% were coordinated actions and about 35% complementary tasks. Therefore, in spontaneous tasks bimanual actions are less common than unimanual actions, and bimanual complementary tasks are the least common. These authors also concluded that bimanual actions seem to be more indicative of hand laterality than unimanual actions.

Recently, two experimental tests were used to reevaluate hand preference at FM, this time with 14 individuals (3 females and 11 males). These two experimental tests were the “simple reaching” and “tube task” tests (Figure 1) [49]. Simple reaching involved simple motor actions and consisted of observing the hand responses of individuals undertaking tasks eliciting fine precise manipulation. The tube task was proposed by Hopkins [69] as a measure to test hand preference because it is a bimanual task sensitive to determining hand motor bias [58, 62]. Simple reaching is actually a unimanual task, while the tube task is a bimanual CRD task as defined by Guiard [57] and Uomini [56].

The simple reaching task was observed daily during midday feeding. In order to encourage the use of fine precise manipulation, every item (peanuts, pieces of apple, muesli, bread, etc.) was smaller than 3 × 3 cm. The procedure consisted of the keeper scattering the food directly on the ground, providing enough food for all the animals in order to prevent any possible dominant-subordinate conflicts. The observation session continued until the subject performed ≥100 simple reaching manual events, as proposed in similar studies [70]. For the tube task, a variant called the “hose task” was designed, in which cylindrical rubber hoses were used in place of rigid tubes. Hoses were filled with honey, peanuts, muesli, and seeds, thus preventing extraction with the tongue or by hitting the hose. The subjects had to remove the food with their fingers or with tools such as sticks or branches. All the individuals (*n* = 14) were evaluated for both experimental tasks (simple reaching and hose task).  Table 1 offers the results about their hand preferences. The experimental protocol and the methodology for data analyses can be consulted in Llorente and colleagues [49].

The results of the simple reaching task showed that 12 individuals were lateralised and 2 were not: 9 (64.29%) were right-handed, 3 (21.43%) were left-handed, and 2 (14.29%) showed no preference. These results are inconsistent with Fagot and Vauclair's suggestion that simple reaching is a low-level task [52], a type of task from which we would expect to see weak evidence of preference.

The results of the hose task showed that all individuals were lateralised. Ten individuals (71.43%) were right-handed and four (28.57%) were left-handed, supporting Hopkins suggestion that chimpanzees may be preferentially right-handed for this type of bimanual (CRD) task [58, 69]. Also, the strength of hand preference was high in the sample, and it did not vary between groups. This may indicate that the hose task elicited a strong lateralisation in individuals [63]. There were no statistically significant differences between the number of right-handed and left-handed subjects, so there was no population level handedness in our chimpanzee sample. 80.64% of the individuals used their fingers (63.83% index finger), while 19.36% of the individuals used tools to extract the food. We detected no differences between digital and tool techniques regarding hand preference: tools seemed to have no effect on the direction of preference, possibly because both left-handed and right-handed individuals used this technique.

Comparing the simple reaching and hose tasks, our results reveal that chimpanzees are right-handed or on the significance borderline for right-hand preference at the population level. This is the first time that chimpanzees housed at a naturalistic environment have yielded this result. Comparing hand preferences for the hose task and for simple reaching, the bimanual task elicited significantly greater individual asymmetries than the simple reaching task, a low-level task. This may be influenced by tool techniques and by the dominance of the index finger as a method of food extraction in the hose task.

Interestingly, we did not detect handedness at the population level in the earlier study at FM in the performance of spontaneous, low-level tasks [65]. Along with the inconsistency between the hose task and simple reaching, this may suggest that hand laterality is a multidimensional trait, as suggested by other authors [31, 71]. In their opinion, motor and neurological demands and requirements are different for these diverse tasks (spontaneous experimental, unimanual, and bimanual coordinated or complementary) [72].

Another interesting feature is that in the hose task 19.36% of subjects used small sticks as tools to access the food, which means that almost 20% of the individuals took on the complex task assisted by a technological behaviour. In contrast, the use of tools in spontaneous behaviour is only present in around 4% of actions.

In summary, our experiments pointed to two main conclusions. On the one hand, at a methodological level, bimanual CRD tasks are not important by themselves, but as part of the wider group to which they belong: the complex tasks, either unimanual or bimanual. However, complementary bimanual tasks appear to be the most complex tasks, since they entail variables such as precise actions, the number of stages required by the task, the number of elements to be combined, the need for using both hands, the sequence of actions, the use of one hand as subordinate, and a complex control of body balance [73–75]. On the other hand, at an evolutionary level complex tasks, as opposed to spontaneous tasks, force the expression and the emergence of hand laterality and technological behaviour.

### 2.2. Chimfunshi Wild Orphanage

As a control measure for the FM experiments, we considered the possibility of replicating the hose task at the Chimfunshi Wild Orphanage (CWO) in Zambia [51] with several naturalistically housed chimpanzees, notably less humanised than the FM individuals.

CWO opened 25 years ago and today shelters 120 chimpanzees, 61 of which were born in captivity and reared by their mothers as in the wild. Most of them were confiscated to prevent the smuggling of infant animals to be later sold as pets or were taken from dilapidated zoos and circuses from all over the world. Their ages range between newborn and 33 years old (see Table  1 in [49], for additional information about age, class, sex and rearing history of each group). Chimpanzees at CWO live in groups in different enclosures, including outdoor enclosures and indoor quarters. The average size of the indoor rooms is 6 × 4 metres. Outdoor enclosures are carved out of the forest and floodplains along the upper Kafue River, with enough thick jungle and fruit groves and open grasslands to allow the chimpanzees to roam almost like in the wild (see [51], for more details).

The aim of our study [51] was to evaluate hand preferences in bimanual complementary actions through observing subjects performing the hose task. We applied the same methodology as used at FM. Out of the 120 individuals in the sample, 100 obtained the minimum number of responses required (*n* = 50) and a minimum of six responses for each test. The experimental protocol and the methodology for data analyses can be consulted in Llorente and colleagues [51].

At CWO the results were similar to those obtained at FM. Overall, a total of 14,854 manual actions were observed: 55.48% (*n* = 8, 241) were performed with the right hand and 44.52% (*n* = 6, 613) with the left hand. Based on binomial tests, 14% of individuals showed no hand preference, and 86% were lateralised for this task: 48 were right-handed and 38 were left-handed. According to the laterality index of the four tests as a whole (see [51], for details on the analytical method), individuals were not lateralised at the population level, although they were at borderline significance. However, when analysing the four tests individually, two tests showed right-handedness at the population level. When analysing only the two first experimental tests (test 1 + test 2), the sample was also clearly right-handed at the population level.

In 95.66% of the actions observed, the subjects removed the food with their fingers (mostly the index finger), and in 4.34% of the actions they used tools. According to our results, subjects performing extractions with the index finger preferentially did so with the right hand, which was consistent with other studies on chimpanzees [69] and other primates [54]. It looks like the use of the index finger as an extracting technique encouraged the use of the right hand. On the other hand, subjects performing extractions with their little finger or tools did so with the left hand. Therefore, a relationship was observed between the use of the little finger, tools, and the left hand, although as yet no explanation for this relationship has been proposed. However, it seems that hand laterality is affected by the distal motions of fingers and hands when performing bimanual complementary tasks in which each hand plays a distinct role. According to Brinkman and Kuypers [76], distal movements require frequent use of the contralateral brain hemisphere, what may explain our results. In addition, the index finger is the most sensitive because it has the largest neuronal representation in motor cortex [77], what may explain its higher use.

Finally, the statistical test used to detect different behaviours between human-reared chimpanzees and mother-reared chimpanzees did not reveal significant differences either in the direction or in the degree of preference. Thus, the original environment and context from where these individuals came did not have any effect on their hand preference patterns. This conclusion had previously been reached by other authors in studies with a sample large and varied enough to test this variable [78]. Actually, these results had also come to light in our earlier study [65], where the observation of hand laterality in the FM chimpanzees at spontaneous unimanual tasks yielded similar results to the wild samples. So, these data seem to indicate that environment cannot explain the disparity of results regarding the current pattern of hand preference in nonhuman primates.

## 3. Technology and Hand Laterality in Human Evolution

Based on the behaviour of great apes [79–83], it is likely that before stone tool manufacture the earliest hominins made use of perishable materials such as sticks and branches and employed materials such as nonmodified bones and stones as tools [84]. It is possible that the first lithic morphotypes were the result of stones being used to crack nuts on anvils, which may have led to accidental flaking, as documented in the Gombe chimpanzees [80] and in Bossou [85]. Some of the flakes with sharp edges may have remained as passive tools until hominins used them to carry out other activities.

As described elsewhere [86], the process of lithic production is derived from objects being used and handled. This adaptive behaviour, which has also been observed in some mammals, birds, and insects, leads to more complex behaviours when the size of the brain increases. Before stone tool production was systematised at African sites, a background would have been in place that facilitated this leap to exosomatic production. As Toth and Schick state [87, page 299], “a decrease in the size of jaws and teeth over time may be correlated with the rise in exosomatic tool use, with technology creating “synthetic organs” and gradually allowing hominins to move into niches traditionally occupied by other animals, such as the carnivore guild.” However, it is not possible in archaeology to identify this basic technological behaviour, or even the manufacture of one simple tool, since such isolated findings are difficult to identify and impossible to classify as intentional. Therefore, it is only possible to identify this process in archaeology when a method of lithic production has been established.

The earliest recorded lithic industry comes from the Ethiopian site of Kada-Gona [88–90], which dates to 2.6 mya. Other sites dated to around 2.4–2.3 mya include Kada-Hadar [91, 92] and Omo-Shungura [93] (both in Ethiopia), Lokalalei (Kenya) [94], and Senga 5A (DR Congo) [95]. The lithic production at these sites was aimed at obtaining flakes with sharp edges, and such artefacts are abundant and diversified, suggesting that the technology was not newly formed [94] but had already been generalised by this time. This means that technology may have originated in Africa some time before this date, perhaps even as early as around 3.5 mya [84, 86, 96]. Recent findings of cut marks on bones at the site of Dikika in Ethiopia [97] confirm this hypothesis.

The archaeo-paleontological scope is rather limited regarding evidence of hand laterality, although not as much as Uomini [56] describes. Actually, hominin hand laterality has been well established for the European *Homo heidelbergensis* of 500,000 years ago [98, 99]. According to our research at Atapuerca (Spain), this hominin species already showed modern-like hand laterality. These results come from two independent sources of evidence: tooth-wear analyses and use-wear traces on tools. Dental microwear analyses have been used to determine hand laterality in hominin species. Since the earliest stages of human evolution, hominins have used their teeth to process their food. Tasks which involved putting the anterior teeth in contact with other materials produced marks and traces on dental surfaces, which are known as dental wear traces of cultural origin. Right-handed individuals and left-handed individuals produce tooth marks oriented in opposing directions. Archaeologically, this tooth wear has been documented in *Homo heidelbergensis* from Sima de los Huesos (Atapuerca, Spain, c. 450 ky) [99], showing the same tendency as in modern humans. On the other hand, use-wear analyses on the edges of the tools made, used, and discarded by the same hominin population (*H. heidelbergensis*) at the site of Galería (Atapuerca, Spain, 400–200 ky) concluded that these tools were used by right-handed individuals [98].

## 4. Discussion

Our results revealed a certain connection between hand laterality, task complexity, and technology. We believe this same connection may apply to human evolution. To trace it back, we have two different groups of data: present-day primates (both human and nonhuman) and archaeological and paleoenvironmental evidence about extinct hominins.

Two fundamental conclusions can be drawn with regard to present-day primates. Firstly, the more complex the task is, the more hand laterality is expressed in humans and apes, regardless of the differences in their brain capacity. Secondly, modern apes mainly show technological behaviour when performing complementary bimanual tasks (CRD).

Regarding hand laterality and task complexity, we believe there is a gradient of manual motor complexity that influences the expression of hand laterality in apes. The more complex the task, the more hand laterality is expressed. Therefore, according to their increasing complexity, tasks would be ordered as follows: (1) unimanual spontaneous tasks, (2) precision-handling (grip) unimanual tasks (such as simple reaching), and (3) bimanual complementary (CRD) tasks, such as nut-cracking and the hose task. Coordinated bimanual tasks (i.e., Uomini's flint puzzle, [56]) are more complex than unimanual tasks and less than CRD tasks, but they are not indicative of handedness in humans and in apes as yet there is no available data. According to our results, the more complex the tasks, the less common they are in the spontaneous behaviour of an individual. Unimanual tasks with no precision grip are the most common tasks, followed by unimanual tasks with precision grip. Finally, the most seldom performed actions are complementary bimanual tasks (CRD tasks).

Present-day humans appear to be ruled by the same gradient of manual motor complexity. Despite the fact that *Homo sapiens *express manual preference even for unimanual tasks with no precision grip, Uomini's research [56] has shown that some tasks do not elicit the expression of hand laterality, while others clearly do. The former are coordinated bimanual tasks (e.g., the flint puzzle) and the latter complementary bimanual tasks (e.g., nut cracking). Although humans have three times the brain capacity of apes and greater brain organisation complexity and are clearly more lateralised animals, they are as prone as apes to this gradient of manual motor complexity. Therefore, when performing simple tasks, *Homo sapiens *elicit a low degree of significant hand laterality. Meanwhile, hand laterality is much more significant when performing complex tasks, as demonstrated by Uomini [56], hence, the complexity of hand laterality tests for humans. Anyone can hold a glass of water with his or her nondominant hand; however, writing with the nondominant hand is almost impossible.

Concerning task complexity and technological behaviour, our results with FM and CWO chimpanzees showed that CRD tasks not only forced the expression of hand laterality but also seem to be behind a higher use of tools. In fact, CRD tasks, the most complex motor tasks, forced the emergence of technological behaviour. Indeed, the concept of maximum complexity would refer to those tasks in which the body itself does not suffice to complete the task at hand. Hence, the correlation evidenced by Schick and Toth between the reduction in the size of the mandibles and teeth in hominins over time and the increase in tool-assisted strategies, developing what they called “synthetic organs” [100, page 299]. Therefore, the manual or functional complexity of the task forces the expression of hand laterality and the emergence of technological behaviour. In modern humans, however, the use of tools is no longer linked to task type. Modern humans use tools to satisfy all tasks, whether simple or complex. Nevertheless, for modern humans and chimpanzees alike, the more complex the task, the more difficult it is to complete without tools.

**Figure 2:**
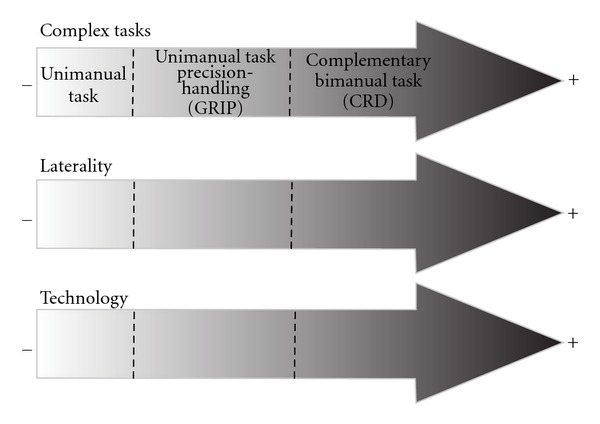
Relationships between complex tasks, hand laterality, and technological behaviour.

With our results from FM and CWO chimpanzees and Uomini's results [56] from modern humans, we aim to approach a particular scenario of hominin evolution with the second group of data: archaeological and paleoenvironmental evidence about extinct hominins. We aim to explore the first interactions between complex tasks, hand laterality, and technological behaviour in human evolution (Figure 2).

To begin with, early hominins such as australopithecines had a cranial capacity that exceeded that of present-day chimpanzees (mean values of 400–500 cm^3^ versus 300–400 cm^3^, resp.) [101]. Therefore, we can assume that they possessed at least the same capacities. It is likely, then, that basic technological behaviours, such as CRD tasks like termite fishing with sticks, would not be unusual among these hominins.

However, unlike chimpanzees, early hominins inhabited an increasingly more arid environment. The close woods that dominated in Africa until ca. 3.5 myr ago were gradually replaced by open forests, savannas, and steppes around 2.5 myr ago. Unlike closed woods, these new landscapes were increasingly unpredictable [96] because resources were more widely dispersed both in terms of space (mosaic) and time (seasonality) [102]. This resource dispersion forced hominins to adopt a generalist diet in order to maximise energy intake ([103], for later species such as *Homo erectus*, [96]).

The adoption of this generalist diet probably involved a diversification of feeding activities, so complex tasks may have become more and more commonplace in this increasingly cruder environment. These conditions may have involved the management of meat, wood, and vegetation, probably requiring cutting actions, which are always CRD tasks. This may be the starting point in human evolution from which cutting tasks become habitual and essential actions. As highlighted by Schick and Toth, cutting tasks are not usually needed in the world of apes [104]. But, cutting actions, like all other CRD tasks, elicit the most both hand laterality and the use of tools.

Therefore, compared to their ancestors, early hominins more frequently practised complex tasks that forced the expression of hand laterality. While their ancestors may have expressed hand laterality only occasionally, like modern nonhuman primates, early hominins would have displayed this trait so often that it would have become permanent. As pointed out by Teixeira and Okazaki [105], there may be a feedback loop involved. The preferential use of one hand would bestow more skill to that hand, increasing the amount of experience provided to that hand and, thus, reinforcing hand laterality. Actually, as some authors have proved, strong individual laterality is associated with increased efficiency in *Gorilla* [106] and *Pan* [107]. In turn, the increase in hand laterality may have favoured an increase in brain laterality, also in feedback loop fashion. The more hand laterality was reinforced, the more the individual was being lateralised for his or her brain functions.

So, we can assume that early hominins such as *Australopithecus afarensis* and *Australopithecus garhi* were already developing technological behaviour and diversifying their diet, as evidenced by the cut marks on herbivore bones at the site of Dikika [97]. This data indicates that these early hominins had already started manufacturing isolated cutting tools around 3.3 mya, one million years prior to the earliest lithic assemblages known to date. They may have regularly practised complex tasks, especially bimanual tasks and particularly CRD tasks, such as cutting. Therefore, the complexity of these tasks forced the expression of hand laterality in these hominins, probably on a regular basis.

What is more, these hominins moved on to technological production while some of their contemporaries maintained the same technological behaviours. Technically, the difference may have lain in the precision and efficiency of percussion, probably enhanced by better defined hand laterality, and in the incorporation of a particular material: stone. At this point the divergence between early hominins and contemporary primates may have broadened, because task acquisition started becoming more and more complex. As pointed out by Byrne [108] from [109], “hammer and anvil use is much slower to acquire than any other manual skill in any ape species.” Actually, according to Schick and Toth [104], apes are poorer stone knappers than early hominins even after years of training. Therefore, Byrne appears to be right when affirming that “the ability to control blows (…) seems to be a crucial adaptation of the human lineage” [108, page 16].

At some point along this process, we find species like *Homo habilis/rudolfensis*. They show a considerable increase in their brain capacity (600–700 cm^3^), as well as in their brain reorganisation [101]. The causes for the increase in brain capacity remain controversial; however, it is likely that there was a constant feedback loop involving hand laterality, complex tasks, technological production, and brain increase, enhanced by meat consumption [110] as part of the generalist diet. A constantly developing brain would benefit the enhancement of operative intelligence, with several consequences. Firstly, hominins became capable of performing increasingly complex tasks more frequently. Secondly, technology was indissolubly established in human evolution. Thirdly, complex tasks were performed through standardised technological behaviour. Fourthly, hand laterality was expressed more often and became permanent. Finally, hominins were able to maximise energy from any resource.

Therefore, the technological scenario of early hominins went from basic technological behaviour to the manufacture of isolated tools and eventually to the establishment of systematic methods of technological production. Eventually, around 2.6 mya, hominins (*Homo habilis/H. rudolfensis*) would establish systematic methods of technological production. These systematic methods would include not only the habit of extracting flakes from cores but also the development of production methods with which to do so (centripetal, unipolar, etc.) and even the method of retouching simple flakes to make shaped tools. All these production tasks are necessarily complementary role differentiation (CRD) tasks, as are most of the processes involved in technology. Over the course of this technological development, hand laterality would have become permanent.

The final consequence of this technological development would be the possibility of maximising the energy intake from any resource. This possibility implies better adaptation to any environment (especially if hominins were permanently assisted by technology) and, therefore, the occupation of new and diversified environments and greater biological development and, consequently, a demographic increase. Actually, social learning and cultural transmission would have probably also developed at the earliest stages of tool manufacture or when lithic production methods were established, in order to socialise the innovations into the community [111], which led to the development of populations.

## 5. Conclusions

Although living environment has been proposed as an important component in explaining the disparity of results regarding hand preferences in chimpanzees, the results of our studies at FM and CWO would reject this hypothesis. The original environment and context from which the animals come do not have any effect on their hand preference patterns.

However, two aspects do seem to be crucial in expressing hand laterality: the type of task being performed and the role performed by the hands during the activity. Our studies confirmed that chimpanzees do not show hand laterality according to activity but may show a low degree according to individual when performing spontaneous *unimanual tasks*, the most common tasks in their daily activities. However, the same individuals displayed higher degree of hand laterality when facing *unimanual tasks that require a precision grip*. Furthermore, *bimanual complementary tasks*, where each hand performed different motions, were infrequent in spontaneous behaviour, but involved the highest degree of hand laterality and the emergence of tool use, as observed during the hose task. Interestingly, although the frequency of tool use varied from FM to CWO chimpanzees, technological behaviour emerged particularly in bimanual complementary tasks (CRD tasks).

Therefore, there appears to be a gradient of task complexity that forces the individual expression of hand laterality and technological behaviour. This gradient would start from spontaneous unimanual tasks, which do not show handedness. Then, we would find the unimanual tasks requiring precision-grip expressing stronger hand laterality. At the extreme of this gradient, there would be the complementary bimanual tasks, such as nut cracking and the tube task.

All processes involved in tool configuration and production are complementary bimanual tasks, as well as most of the subsistence activities carried out by the earliest hominins (and also by modern humans). The need to maximise the supply of energy in an unpredictable landscape forced early hominins to increase the number and complexity of the subsistence activities performed daily. Therefore, previously infrequent complementary bimanual tasks became almost permanent. This, in turn, forced the frequent expression of hand laterality and technological assistance, which had up to then been quite uncommon. As this expression developed, the efficiency of the dominant hand also developed, as well as the efficiency of the tools produced. Hence, this constant loop led to the gradual complexity of the tasks performed, the gradual implementation of hand laterality, and the development of technological support, which in turn favoured the development of the brain motor and associative areas concerned. From this point onwards, brain, technology, and hand laterality were involved in a continuous feedback loop.

## Figures and Tables

**Table 1:** Hand preferences and consistency for *simple reaching* and *hose task* at the FM chimpanzees. R: right-hand preference. L: left-hand preference. A: Nonpreferent.

Subject	Hand preference	Hand preference	Consistency
	*Simple reaching*	*Hose task*	
Bongo	R	R	Yes
Charly	L	R	No
Julio	L	R	Yes
Juanito	A	R	No
Marco	R	R	Yes
Nico	R	R	Yes
Pancho	R	R	Yes
Romie	L	L	Yes
Sara	R	L	No
Tico	R	L	No
Toni	L	R	No
Toto	R	R	Yes
Victor	R	R	Yes
Waty	A	L	No
